# A multimodal atlas of tumour metabolism reveals the architecture of gene–metabolite covariation

**DOI:** 10.1038/s42255-023-00817-8

**Published:** 2023-06-19

**Authors:** Elisa Benedetti, Eric Minwei Liu, Cerise Tang, Fengshen Kuo, Mustafa Buyukozkan, Tricia Park, Jinsung Park, Fabian Correa, A. Ari Hakimi, Andrew M. Intlekofer, Jan Krumsiek, Ed Reznik

**Affiliations:** 1 Department of Physiology and Biophysics, Weill Cornell Medicine, New York, NY USA; 2 Institute of Computational Biomedicine, Weill Cornell Medicine, New York, NY USA; 3 Computational Oncology Service, Memorial Sloan Kettering Cancer Center, New York, NY USA; 4 Department of Surgery, Urology Service, Memorial Sloan Kettering Cancer Center, New York, NY USA; 5 Human Oncology and Pathogenesis Program, Memorial Sloan Kettering Cancer Center, New York, NY USA; 6 Englander Institute for Precision Medicine, Weill Cornell Medicine, New York, NY USA; 7 Center for Molecular Oncology, Memorial Sloan Kettering Cancer Center, New York, NY USA

**Keywords:** Cancer metabolism, Data integration, Metabolism, Computational biology and bioinformatics

## Abstract

Tumour metabolism is controlled by coordinated changes in metabolite abundance and gene expression, but simultaneous quantification of metabolites and transcripts in primary tissue is rare. To overcome this limitation and to study gene–metabolite covariation in cancer, we assemble the Cancer Atlas of Metabolic Profiles of metabolomic and transcriptomic data from 988 tumour and control specimens spanning 11 cancer types in published and newly generated datasets. Meta-analysis of the Cancer Atlas of Metabolic Profiles reveals two classes of gene–metabolite covariation that transcend cancer types. The first corresponds to gene–metabolite pairs engaged in direct enzyme–substrate interactions, identifying putative genes controlling metabolite pool sizes. A second class of gene–metabolite covariation represents a small number of hub metabolites, including quinolinate and nicotinamide adenine dinucleotide, which correlate to many genes specifically expressed in immune cell populations. These results provide evidence that gene–metabolite covariation in cellularly heterogeneous tissue arises, in part, from both mechanistic interactions between genes and metabolites, and from remodelling of the bulk metabolome in specific immune microenvironments.

## Abstract (web summary)

Benedetti et al. produce a Cancer Atlas of Metabolic Profiles from 988 metabolomic and transcriptomic tissue profiles across 15 studies to analyse and identify gene–metabolite interactions that transcend cancer types.

## Main

Coordinated changes of genetically encoded metabolic enzymes and transporters, and the metabolites they act on, underpin diverse cancer-associated phenomena, including tumorigenesis^1^, pluripotency^2,3^, the onset of drug resistance^4–6^ and the modulation of immune responses^7–11^. However, despite the high value of joint profiling of metabolites and gene expression/protein levels, previous large-scale studies of tumour metabolism have overwhelmingly focused on the analysis of gene expression data^12^. Conversely, the few instances of multimodal metabolomic and transcriptomic profiling of human tumour specimens have largely been performed in disparate, unrelated studies by a multitude of research teams^13–22^. Integration of metabolomic datasets produced in different patient cohorts is challenging due to technical batch effects and the semi-quantitative nature of untargeted metabolomic data (reported in arbitrary units of relative abundance). Thus, both the scarcity of multimodal metabolomic/transcriptomic data from tissue specimens and the challenges of harmonizing available datasets fundamentally impede the discovery of recurrent, coordinated changes in metabolic gene expression and metabolite abundance across cancers.

Tumours from diverse cancer types differ in their cell-type composition, vascularization and other factors ultimately influencing metabolism. Yet, they share a convergent set of metabolic alterations^23–27^. For example, several meta-analyses of the tumour metabolic transcriptome have identified recurrent upregulation of genes in one-carbon metabolism and oxidative phosphorylation across cancer types^26,28,29^. Analogously, meta-analyses of metabolomics data have demonstrated that numerous central carbon metabolites (for example, lactate) and effector metabolites (for example, kynurenine) are at higher abundance in tumour tissue compared to normal tissue across many cancer types^23,30^. These studies have illustrated the power of meta-analysis for distilling highly recurrent metabolic phenotypes from heterogeneous data but have left unresolved the question of how metabolic gene expression and metabolite abundance are coordinated and ultimately shape tumour physiology.

To systematically investigate gene–metabolite covariation in cancer, we assembled, harmonized and integratively analysed metabolomics and transcriptomics profiles from 988 primary tumour and matched adjacent normal tissue collected in 15 independent studies covering 11 cancer types. The preprocessed and harmonized data constitute the Cancer Atlas of Metabolic Profiles (CAMP), representing what is, to our knowledge, the largest harmonized dataset of multimodal metabolomic and transcriptomic data on primary tumour specimens. The CAMP is publicly available for download on Zenodo (10.5281/zenodo.7150252) and can be interactively explored at https://rezniklab.shinyapps.io/CAMP-shiny-app/. Leveraging the diversity of diseases in our dataset, we designed a concordance-based statistical meta-analysis approach to discover instances of gene–metabolite interactions (GMIs) that transcended cancer type. This revealed two distinct classes of GMIs: First, we identified a small number of strong interactions between enzymes and metabolites involved in the same or subsequent reactions (‘proximal’ GMIs), suggesting that these enzymes are the primary determinants of their respective metabolite pool sizes. A second group of GMIs consisted of a small number of metabolites broadly correlated to large numbers of genes. Interestingly, this second class of GMIs was enriched for genes specifically expressed in immune cells, and for metabolites related to nicotinamide adenine dinucleotide (NAD^+^), a pleiotropic metabolite that acts both as a central cofactor in metabolism^31^ and as a signalling molecule influencing cell identity^32^. Taken together, these findings suggest that gene–metabolite covariation in tumours emerges, in part, from two complementary phenomena: the expression of enzymes with strong control over metabolite pool size, and the presence of specific cell populations in the tumour microenvironment with characteristic metabolomic profiles.

## Results

### The Cancer Atlas of Metabolic Profiles

**Fig. 1:**
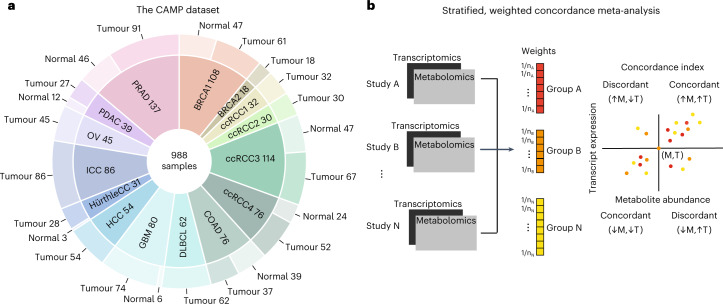
Summary of the Cancer Atlas of Metabolic Profiles. **a**, The CAMP integrates metabolomic and transcriptomic data from 15 datasets covering 11 different cancer types, comprising both tumour and normal tissue. BRCA1/BRCA2, breast cancer; COAD, colon adenocarcinoma; DLBCL, diffuse large B cell lymphoma; GMB, glioblastoma; HürthleCC, Hürthle cell carcinoma; OV, high-grade serous ovarian cancer; PDAC, pancreas adenocarcinoma; PRAD, prostate adenocarcinoma; ccRCC1/ccRCC2/ccRCC3/ccRCC4, clear-cell renal carcinoma. **b**, Overview of non-parametric concordance meta-analysis. For a given transcript (T) and metabolite (M) pair, all measurements from all datasets were considered and weighted according to sample size. The concordance is a non-parametric measure of bivariate association between T and M that can be applied in meta-analysis across multiple datasets. Source data

**Extended Data Fig. 1:**
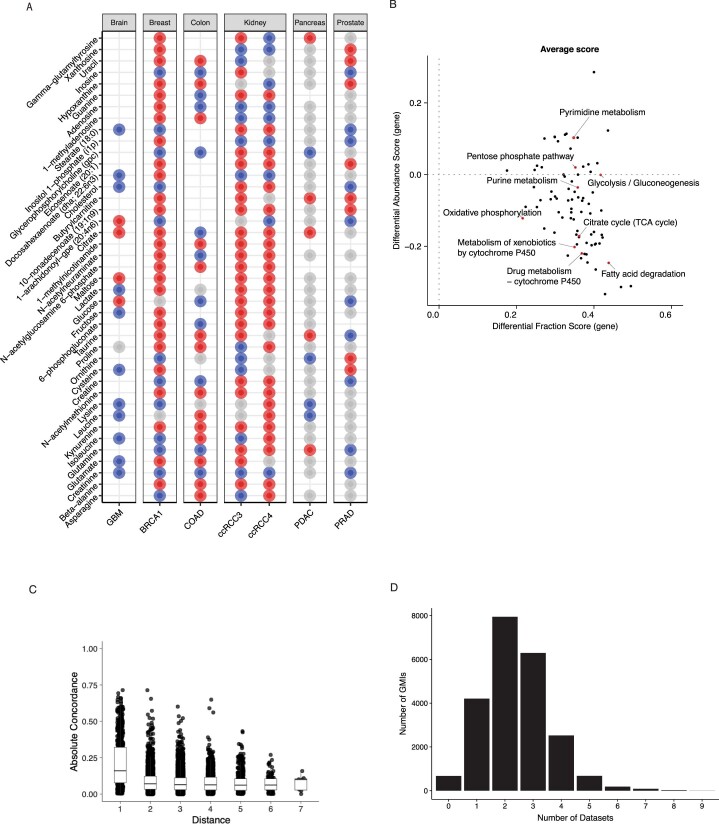
The Cancer Atlas of Metabolic Profiles (CAMP) data recapitulate prior work interactions and demonstrate robust GMIs. (A)Differential abundance between tumor and normal samples across 7 cancer types. Blue circles indicate metabolites depleted in tumor related to normal tissue and red circles indicate increased in tumor related to normal tissue. Grey circles mean metabolites with no statistically significant change in abundance. (B) Scatterplot of gene expression differential fraction score and differential abundance score in the KEGG metabolic pathways. (C) Boxplot comparing absolute concordance to metabolic distance from 159 metabolites that with KEGG id in the human 1 GEM model (12560 pair-wise distance). The data is displayed as a boxplot, in which the box bounds represent the 25th to the 75th percentile, with the median shown as a line in the center. The bottom whisker extends to the smallest value that is not an outlier, and the upper whisker extends to the largest value that is not an outlier. (D) Barplot of statistically significant GMIs and the number of individual datasets the GMI is significant in after FDR correction. Source data

Since metabolomic profiling has so far been excluded from large multimodal tumour profiling projects (for example, the TCGA^33^), there is no unified resource of metabolomic/transcriptomic data in the cancer research field. However, several groups have independently produced and released matched metabolomic/transcriptomic data in diverse cancer types^13–22^. We combined these datasets with several in-house studies to create a comprehensive collection of 988 samples (764 tumour samples and 224 adjacent normal samples) across 11 different cancer types, covering 15 datasets, which we called the CAMP (Table 1 and Fig. 1a). The overall collection includes a total of more than 40,000 unique transcripts and almost 2,500 unique metabolites. To maximize comparability across these heterogeneous studies, we applied a unified workflow to process RNA expression from microarray and RNA-sequencing (RNA-seq) data, harmonize metabolite names and annotations, and standardize data normalization and preprocessing (Methods). The CAMP represents an unprecedented resource to investigate the covariation of metabolite levels and gene expression at scale across diverse lineages of human cancers and normal tissues. To ensure high quality of the data, we evaluated several measures of quality control (QC). These included confirming that changes in metabolite abundance and gene expression between tumour and normal samples recapitulated those from prior work (Extended Data Fig. 1a,b), and demonstrating that covariation between pairs of metabolites was strongest between proximal metabolites in the metabolic network (that is, metabolite pairs acted upon by a common enzyme; Extended Data Fig. 1c).

**Table 1:** Overview of cancer type, sample size and type of gene expression data in the study

Cohort	Cancer type	Reference	Samples (tumour/normal)	Gene expression data type
BRCA1	Breast cancer	Terunuma et al.^19^	61/47	Microarray
BRCA2	Breast cancer	Tang et al.^20^	18/–	RNA-seq
COAD	Colon adenocarcinoma	Satoh et al.^18^	37/39	Microarray
DLBCL	Diffuse large B cell lymphoma	Calvo-Vidal et al.^17^	62/–	RNA-seq
GBM	Glioblastoma	Wang et al. ^16^	74/6	RNA-seq
HürthleCC	Hürthle cell carcinoma	Ganly et al. ^84^	28/3	RNA-seq
HCC	Hepatocellular carcinoma	Chaisaingmongkol et al. ^15^	54/–	Microarray
ICC	Intrahepatic cholangiocarcinoma	Chaisaingmongkol et al. ^15^	86/–	Microarray
OV	High-grade serous ovarian cancer	Gentric et al. ^14^	45/–	Microarray
PDAC	Pancreas adenocarcinoma	Zhang et al. ^21^	27/12	Microarray
PRAD	Prostate adenocarcinoma	Penney et al. ^13,85^	91/46	Microarray
ccRCC1	Clear-cell renal carcinoma	Hakimi et al. ^22^	32/–	RNA-seq
ccRCC2	Clear-cell renal carcinoma	Golkaram et al. ^86^	30/–	RNA-seq
ccRCC3	Clear-cell renal carcinoma	Golkaram et al. ^86^	67/47	RNA-seq
ccRCC4	Clear-cell renal carcinoma	Golkaram et al. ^86^	52/24	RNA-seq

**Fig. 2:**
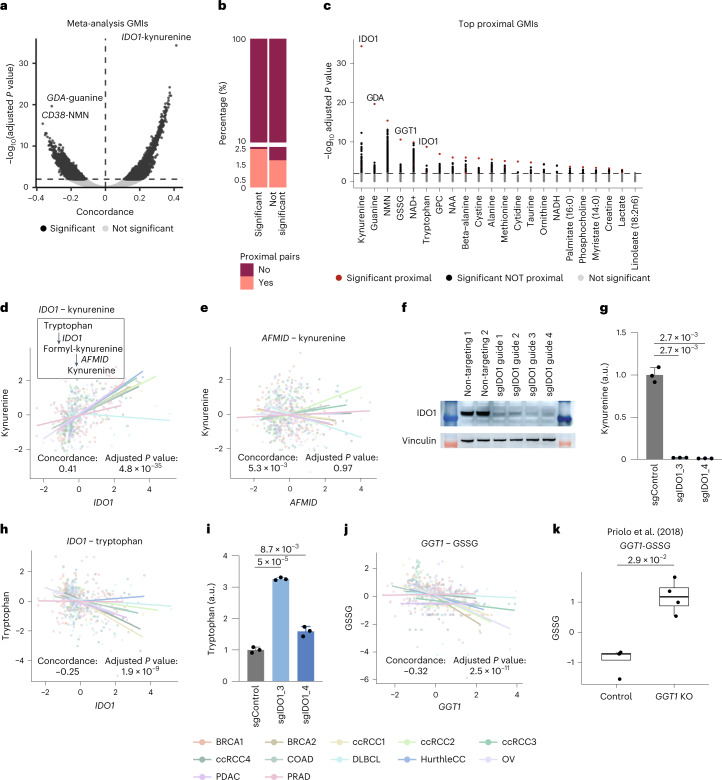
Meta-analysis across the CAMP captures lineage-transcending gene–metabolite interactions. **a**, Volcano plot of the GMIs computed between the 16,082 genes present in all datasets, and the 276 metabolites present in at least 8 of our 15 tumour cohorts. The *x* axis indicates the scaled concordance value, where values above 0 indicate positive association and values below 0 indicate negative association. The *y* axis represents the corresponding −log_10_ FDR-adjusted *P* value. Two-tailed *P* values were estimated from the unscaled concordance value’s *z*-score (Methods) and were corrected for multiple testing using the Benjamini–Hochberg method. The horizontal line indicates the significance cut-off of 0.01 FDR. Light grey dots indicate nonsignificant gene–metabolite pairs, and black dots indicate significant pairs. Three top hits have been highlighted. **b**, Statistically significant GMIs are enriched for proximal interactions, but proximal interactions nevertheless constitute a minority of all statistically significant GMIs. Of all significant gene–metabolite pairs in our concordance meta-analysis, 3,304/22,619 pairs had a defined distance (~14.61%), but only 565/22,619 (~2.50%) of these were proximal. **c**, Proximal GMI prioritization. GMIs for the 22 metabolites whose strongest GMI was proximal (distance less or equal 2). For each metabolite, we ranked genes by their statistical significance. Two-tailed *P* values were estimated from the unscaled concordance value’s *z*-score (Methods) and were corrected for multiple testing using the Benjamini–Hochberg method. Red and black dots indicate proximal and non-proximal genes significantly associated with the corresponding metabolite, respectively, while grey dots indicate genes with nonsignificant associations. Bold metabolites exhibit a large gap between the dominant GMI and all other GMIs for a metabolite. **d**,**e**, Scatterplots of the association between kynurenine levels and two proximal genes (*IDO1*, **d**; *AFMID*, **e**). Metabolite abundances were scaled within each dataset to be displayed together. Two-tailed *P* values were estimated from the unscaled concordance value’s z-score (Methods) and were corrected for multiple testing using the Benjamini–Hochberg method. **f**, CRISPR–CAS9-mediated knockout of *IDO1* depleted IDO1 protein levels in HCT116 cells. Western blot was performed once and not repeated. **g**, Kynurenine levels were depleted upon knockout of *IDO1* in HCT116 cells (*n* = 3 in each condition). Data are presented as mean values ± s.d. **h**, Scatterplot of the association between tryptophan levels and *IDO1* in the CAMP. Two-tailed *P* values were estimated from the unscaled concordance value’s *z*-score (Methods) and were corrected for multiple testing using the Benjamini–Hochberg method. **i**, Tryptophan levels increase upon *IDO1* knockout (*n* = 3 in each condition). Data are presented as mean values ± s.d. **j**, Scatterplot of oxidized glutathione (GSSG) and *GGT1*. Two-tailed *P* values were estimated from the unscaled concordance value’s *z*-score (Methods) and were corrected for multiple testing using the Benjamini–Hochberg method. **k**, Validation of the GSSG and *GGT1* relationship was based on the study from Priolo et al.^42^. The dataset includes four data points for each condition. The two-tailed *P* value was estimated with a Wilcoxon rank-sum test. a.u., arbitrary units. Source data

**Extended Data Fig. 2:**
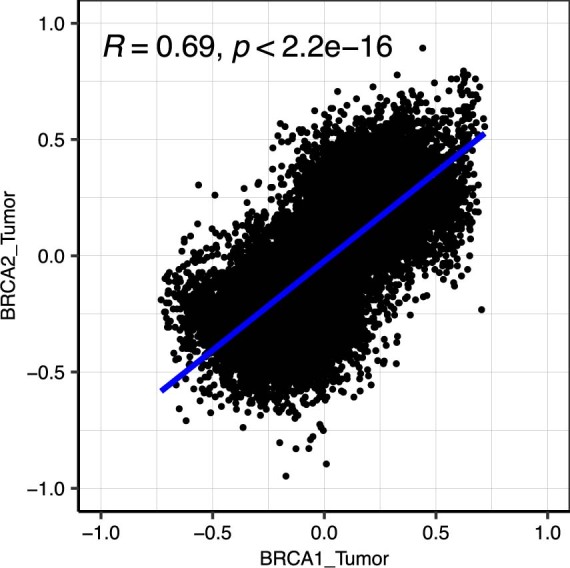
The statistically significant GMIs identified from different transcriptomic profiling technology (microarray v.s. RNA sequencing) were highly consistent. Scatterplot of statistically significant GMIs in BRCA1 (microarray) and BRCA2 (RNA sequencing) P-values was estimated from Pearson’s correlation test. Axes represent concordance values. Source data

We interrogated the CAMP dataset for recurrent covariation between genes and metabolites across datasets and cancers. Such gene–metabolite covariation could emerge via numerous mechanisms including direct metabolic interactions, for example, via expression changes of a rate-limiting enzyme, or by the accumulation/depletion of metabolites as part of a broader phenotype, such as a cytotoxic immune response^34–36^. While each cancer type is likely to demonstrate its own unique pattern of transcriptomic and metabolomic changes, the CAMP enables discovery of metabolomic/transcriptomic covariation that transcends diseases. To identify cancer-type-agnostic metabolite and transcript covariation across CAMP datasets in a statistically principled manner, we developed a concordance-based meta-analysis approach (Fig. 1b and Methods). Concordance is a non-parametric measure of correlation, which enables the identification of consistently positive or negative gene–metabolite associations across datasets^37^. Our measure of concordance ranges from –1 to 1 and is closely related to non-parametric correlation coefficients such as Kendall’s tau, with a value of –1 corresponding to strong discordance and 1 corresponding to strong concordance (Methods). We focused our analysis on tumour samples only in the CAMP (although analogous analysis could be carried out on normal samples), and focused on the 276 metabolites that were quantified in more than half of the tumour datasets (at least 8 of 15 tumour datasets) and the 16,082 genes that were quantified in all 15 studies. Of all possible gene–metabolite pairs (276 metabolites × 16,082 genes = 4,438,632 pairs), a total of 22,619 pairs (0.51%) were significantly correlated after multiple-testing correction at a false discovery rate (FDR) of 0.01 (Fig. 2a). This included 269 metabolites (~97%) and 7,987 genes (~50%) participating in at least one significant association (Supplementary Table 1), which we refer to as GMIs. Post-hoc QC confirmed that 7,737/22,619 (78%) of GMIs were statistically significant in two or more individual datasets (Extended Data Fig. 1d). Statistically significant GMIs identified in single-study concordance analysis of the BRCA1 (microarray) and BRCA2 (RNA-seq) were highly consistent, indicating that the choice of transcriptomic profiling technology (that is, microarray versus RNA-seq) did not introduce substantial artefacts (Extended Data Fig. 2). Importantly, the results of the concordance were not affected by imputation, and an analogous concordance analysis omitting all imputed data reproduced 20,291/22,619 (89.7%) of statistically significant GMIs. Finally, we examined the consistency of GMIs (considering only those measured in >7 distinct datasets) generated from two distinct, non-overlapping subsamples of the full CAMP dataset. This analysis identified a high degree of consistency between estimates of concordance for each GMI (Spearman rho 0.31, *P* value < 10^−^^16^), confirming the robustness of the results of our concordance meta-analysis.

### A subset of gene–metabolite covariation represent direct enzyme–substrate interactions

**Extended Data Fig. 3:**
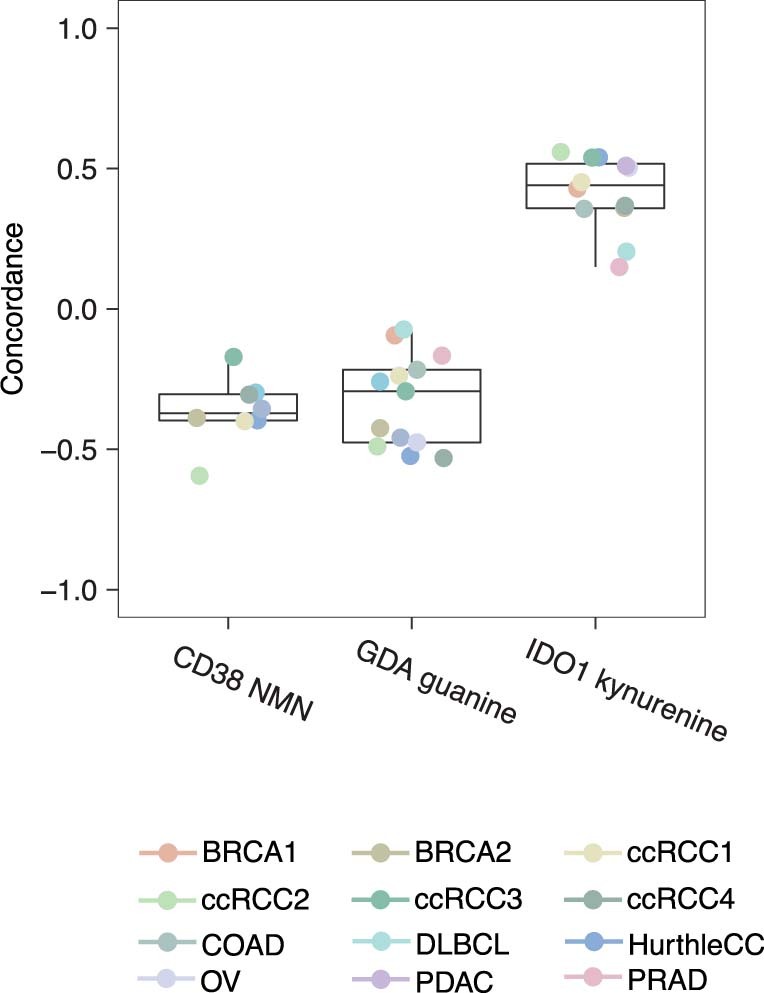
Statistical significance of these 3 GMIs was likely driven by several cancer types. Box plots of concordance for gene-metabolite pair of *CD38*-MNM (n=8), *GDA*-guanine (n=13) and *IDO1*-kynurenine (n=12) across individual datasets. The data is displayed as a boxplot, in which the box bounds represent the 25th to the 75th percentile, with the median shown as a line in the center. The bottom whisker extends to the smallest value that is not an outlier, and the upper whisker extends to the largest value that is not an outlier. Source data

Among the statistically significant GMIs (Fig. 2a), we noted that the strongest positively correlated GMI (*IDO1–*kynurenine, adjusted *P* value = 4.82 × 10^−^^35^) and the two strongest negatively correlated GMIs, *GDA*–guanine (adjusted *P* value = 2.31 × 10^−^^20^) and *CD38*–nicotinamide mononucleotide (NMN; adjusted *P* value = 3.90 × 10^−^^16^) corresponded to ‘proximal’ metabolic interactions, which are interactions between an enzyme and its direct or nearly direct substrate/product. For example, *IDO1* catalyses the catabolism of tryptophan to *N*-formyl-kynurenine, which is subsequently metabolized to kynurenine^38^, and both *CD38* and *GDA* directly degrade the metabolites guanine and NMN, respectively^39,40^. We confirmed that statistical significance for these three GMIs was likely driven by several cancer types rather than a single dataset with very strong associations (Extended Data Fig. 3).

The direct biochemical relationship between the three GMIs above raised the possibility that functional proximity between enzymes and their substrates/products might underlie a large fraction of GMIs. To test this, we systematically computed the biochemical distance between all gene–metabolite pairs using the highly curated Human-1 metabolic network model from Robinson et al.^41^. In this framework, a distance of one represents molecules that are involved in the same metabolic reaction, and a distance of two indicates a gene–metabolite pair that take part in subsequent reaction steps (Methods). Although statistically significant GMIs were enriched for proximal interactions relative to nonsignificant gene–metabolite pairs (odds ratio 1.42, Fisher’s exact test *P* value = 3.09 × 10^−^^15^), proximal interactions themselves constituted only a small fraction of the total ensemble of statistically significant GMIs (2.5%, 565/22,619; Fig. 2b). Thus, while several of the strongest GMIs arose from proximal interactions, gene–metabolite proximity was a weak determinant of the full GMI landscape.

**Extended Data Fig. 4:**
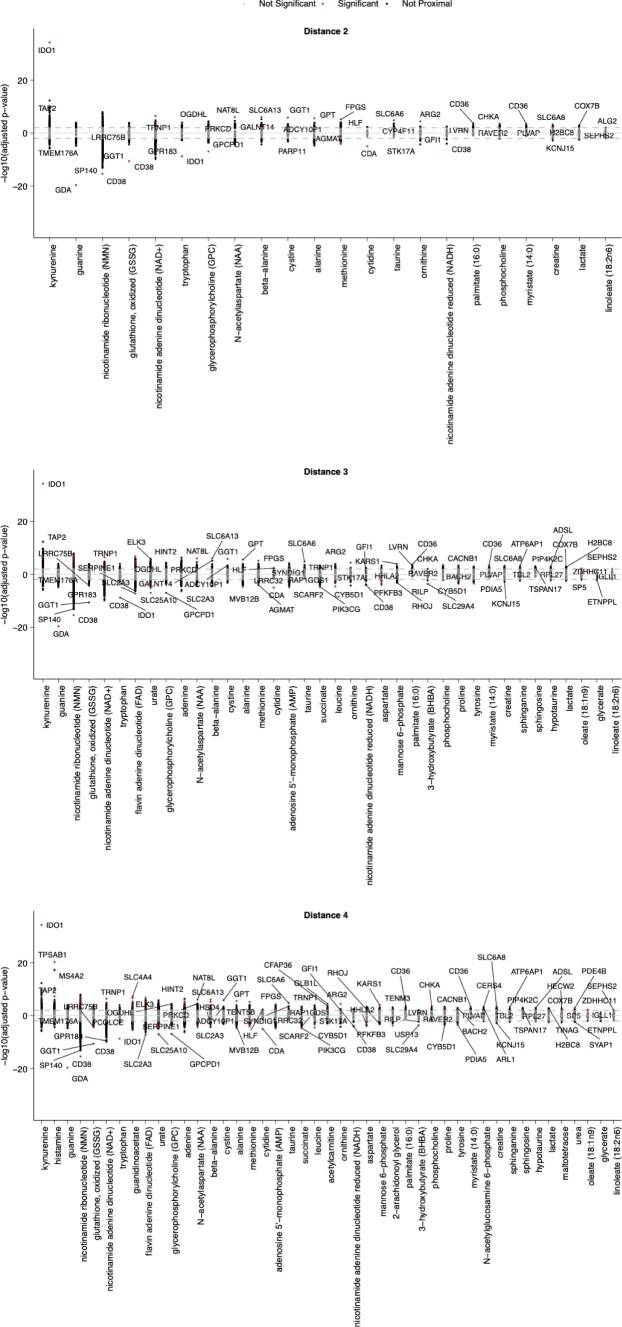
Gene-metabolite proximity at relaxed distance thresholds has similar results. -Log_10_ adjusted p-value of GMIs when definition of proximity is 2 (top), and when it is relaxed to 3 (middle) and 4 (bottom). Two-tailed p-values were estimated from the unscaled concordance value’s z-score (see Methods) and were corrected for multiple testing using the Benjamini-Hochberg method. Adjusted p-values are multiplied by concordance sign, thus values below 0 indicate negative covariation. Source data

To further investigate the above observations about the strength of specific proximal GMIs and the overall relationship between metabolic proximity and GMIs, we investigated the relative strength of different GMIs affecting a common metabolite. We then focused on the 22 metabolites whose strongest GMI was proximal, covering diverse molecules involved in nucleotide metabolism (guanine, cytidine), cofactor metabolism (NAD^+^), redox metabolism (cystine, oxidized glutathione (GSSG)) and other pathways (Fig. 2c). Interestingly, we found that for 8 of 22 metabolites (kynurenine, guanine, NMN, GSSG, tryptophan, glycerophosphocholine (GPC), cystine and cytidine), a large gap existed between the most significant GMI and the second-highest correlating transcript. This gap suggested that the pool size of these metabolites was strongly controlled, in a lineage-agnostic manner, by a single, dominant gene. Consequently, we hypothesized that targeted genetic knockdown of dominant GMIs for these 8 metabolites would have a higher likelihood of producing significant changes in pool size than for other metabolites with multiple, comparably strong GMIs, each of which might control the pool size of the metabolite (Fig. 2c). Similar results were found when relaxing the threshold for calling gene–metabolite proximity (Extended Data Fig. 4).

**Extended Data Fig. 5:**
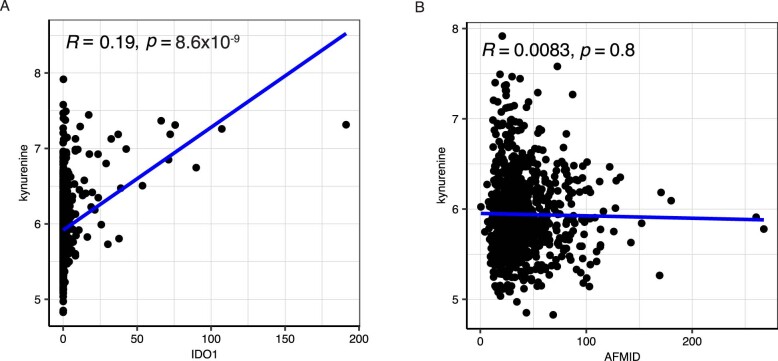
*IDO1*-kynurenine and *AFMID*-kynurenine correlations from the CCLE consortium. (A) Scatterplots comparing kynurenine abundance to *IDO1*, and (B) *AFMID* expression in independent metabolome and transcriptome data obtained from the CCLE consortium. P-value was estimated from Spearman’s rank correlation test. Source data

We sought to functionally validate a subset of these predicted, metabolite pool-controlling genes. First, we investigated the association between *IDO1* and two metabolites, kynurenine and tryptophan. IDO1 converts tryptophan to *N*-formylkynurenine, which is subsequently catabolized to kynurenine by AFMID. We observed that kynurenine levels were strongly associated with *IDO1* (Fig. 2d) but not *AFMID* (which acts directly to produce kynurenine; Fig. 2e) expression across the CAMP. These findings were consistent with independent measurements of the metabolome and transcriptome obtained from the Cancer Cell Line Encyclopedia consortium on ~900 cell lines, where *IDO1* expression was associated with kynurenine abundance (*P* = 8.6 × 10^−^^9^) but *AFMID* expression was not (*P* = 0.8) (ref. ^36^) (Extended Data Fig. 5a,b). To experimentally test the hypothesis that disruption of *IDO1* impacts both tryptophan and kynurenine pool sizes, we used CRISPR–Cas9-mediated knockout with single-guide RNAs (sgRNAs) targeted against *IDO1* human colorectal carcinoma HCT116 cells. These experiments corroborated earlier data indicating that knockout of *IDO1* depleted kynurenine pools (Fig. 2f,g). However, while the association between *IDO1* and kynurenine has been widely described in the literature^36^, our analysis indicated that *IDO1* is also expected to determine the pool size for tryptophan, an amino acid involved in numerous other reactions in the cell, most obviously the synthesis of proteins (Fig. 2h). Consistent with this observation, we observed that knockout of *IDO1* was sufficient to increase tryptophan levels, indicating that the pool size of this highly connected proteinogenic amino acid could be perturbed in part through disruption of IDO1 activity (Fig. 2i). Second, in support of a proximal GMI between *GGT1* and GSSG (Fig. 2j), we reanalysed existing metabolomic data from a functional knockdown of *GGT1* versus control in human embryonic kidney HEK293T cells^42,43^. This data confirmed that knockdown of *GGT1* was associated with an increase in GSSG levels with respect to mock control (*P* value = 2.90 × 10^−^^2^), suggesting that *GGT1* is a pool-determining consumer of GSSG (Fig. 2k). Taken together, these data demonstrate that lineage-transcending GMIs discovered through pathway-based analysis of the CAMP represent examples of genes exerting strong control over metabolite pool sizes.

### Pathway-level metabolic and transcriptomic changes weakly covary

Despite the interesting findings related to proximal GMIs, most (97.5%) statistically significant GMIs represented distant, non-proximal interactions beyond obvious enzyme–substrate metabolic relationships (Fig. 2b). One possible implication of such nonlocal covariation is that genes and metabolites in the same metabolic pathway would show asynchronous and uncorrelated changes across different groups of samples, such as tumours and normal tissues. To investigate this hypothesis, we studied the consistency of transcriptional and metabolic differences in tumour versus adjacent normal tissue across cancer types. To this end, we performed differential analysis of metabolite and transcript levels between tumour and normal tissues in the 7 CAMP datasets where both tissues were available (Table 1) and aggregated the results into 85 Kyoto Encyclopedia of Genes and Genomes (KEGG) metabolic pathways. Of these, we considered 63 pathways with at least one metabolite or gene measured in at least 5 of the 7 CAMP datasets (Supplementary Table 2).

**Fig. 3:**
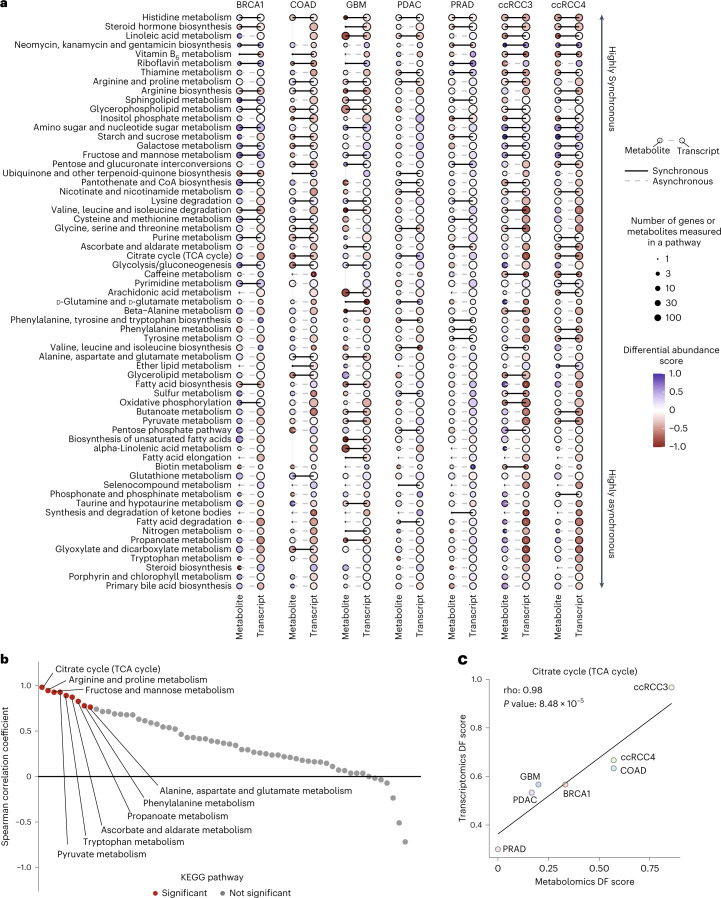
Tumour versus normal changes in metabolite and transcript abundance are predominantly asynchronous. **a**, Heat map of metabolite and transcript DA scores across datasets and pathways, capturing the tendency for metabolites and genes to accumulate or deplete in tumours relative to normal tissues. The size of the dots indicates the number of molecules measured in that pathway, while the colour represents the DA score. **b**, Spearman correlation coefficients of the metabolite and transcript DF scores in KEGG pathways. Red dots indicate nominal significance (*P* value < 0.05). A minority of pathways showed significant association between metabolomic and transcriptomic disruption across CAMP datasets. *P* values were estimated from Spearman’s rank correlation test and were corrected for multiple testing using the Benjamini–Hochberg method. **c**, Example of Spearman correlation calculation: Distribution of the metabolite (*x* axis) and transcript (*y* axis) DF scores across datasets for the citrate cycle (tricarboxylic acid (TCA) cycle) pathway. *P* values were estimated from Spearman’s rank correlation test. Source data

For each KEGG pathway, we evaluated (using a differential abundance (DA) score; Methods) whether metabolites and transcripts showed synchronous accumulation or depletion patterns in tumours relative to normal tissues. Pathways were biased towards asynchronous changes (276/441, 63%), where increases in metabolite levels coincided with decreases in transcript levels, and vice versa (Fig. 3a). Only one pathway (histidine metabolism) demonstrated fully synchronous changes in all datasets, whereas a few others demonstrated uniformly asynchronous changes (for example, primary bile acid biosynthesis). We also assessed whether there was a correlation between the extent of metabolomic versus transcriptomic disruption regardless of the direction (using a differential fraction (DF) score; Methods). A minority of pathways (9/63) demonstrated significant associations (nominal *P* value < 0.05) between RNA and metabolite DF scores (Fig. 3b; see Fig. 3c as an example). Interestingly, these 9 pathways belonged to just 2 KEGG pathway classes (Supplementary Table 3): amino acid metabolism and carbohydrate metabolism. Enrichment analysis indicated that the class of carbohydrate metabolism pathway (of 8 in total) was significantly over-represented relative to the others (Fisher’s exact test *P* value: 2.36 × 10^−^^2^). Thus, most pathways showed no evidence of a correlation between metabolomic and transcriptomic disruption, emphasizing the implications of predominantly distally acting GMIs and prompting the question of which biological phenomena produce these distal GMIs.

### Hub metabolites are enriched for immune genes associations

**Fig. 4:**
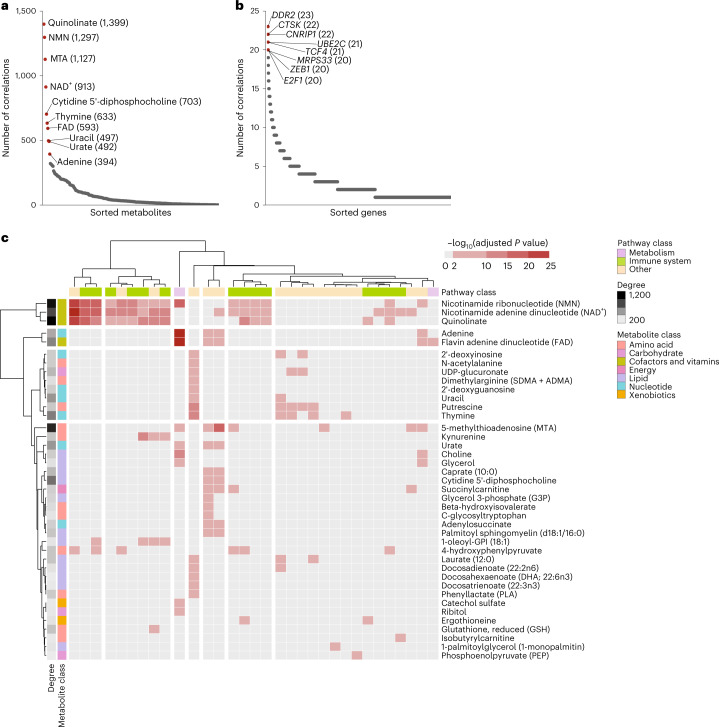
Gene–metabolite interactions are concentrated in hub metabolites associated with immune-related genes. **a**, Distribution of significant GMIs across metabolites. The top ten metabolites with the highest number of significant associations are labelled. **b**, Distribution of significant GMIs across genes. The top eight genes with the highest number of significant associations are labelled. **c**, KEGG-based gene pathway enrichment analysis results for metabolites with at least one GMI and at least one significantly enriched pathway. The heat map colour represents the strength of the enrichment as the negative log_10_(*P* value) of the pathway enrichment test. Two-sided *P* values were estimated with a Fisher’s exact test and adjusted for multiple testing using the Benjamini–Hochberg method. Cells coloured in shades of red indicate pathways that were significant after multiple-testing corrections (adjusted *P* value < 0.01), and grey cells indicate insignificant associations. Individual pathways were classified based on the type of process they describe: metabolism (lilac), immune (green) or other (cream). MTA, 5′-methylthioadenosine. Source data

**Extended Data Fig. 6:**
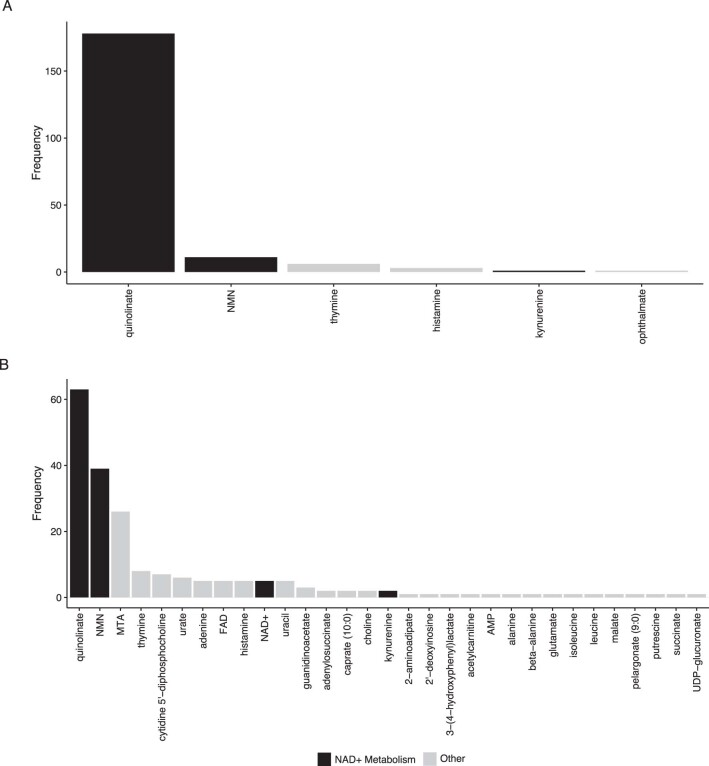
200 strongest non-proximal GMIs. Barplot of metabolite involved in the 200 strongest non-proximal GMIs excluding pairs without a distance (A) and including pairs without a distance (B). Pairs without a distance are denoted as NA in Supplementary Table 1. Metabolites in black belong to the NAD+ metabolism pathway while all others are in grey. Source data

Having established that most GMIs do not represent biochemically proximal interactions, we adopted a broader approach to identify the driving factors of metabolite–transcript correlations. First, we investigated the distribution of GMIs across metabolites and genes, observing that GMIs were strongly concentrated in a small number of metabolites (Fig. 4a). The top three metabolites with the highest number of GMIs, quinolinate, NMN and 5′-methylthioadenosine alone contributed to 17% (3,823/22,619) of all GMIs in our analysis, and the top ten metabolites covered 35% of the GMIs (8,048/22,619), far higher than the fraction covered by the top eight genes (Fig. 4b and Extended Data Fig. 6a,b). That is, a small number of metabolites participated in an exceptionally high number of GMIs, acting as ‘hubs’ for strong covariation with gene expression. Interestingly, hub metabolites concentrated in certain metabolic pathways. Among the top ten most correlated metabolites, we found several constituents of the NAD^+^ biosynthesis pathway (quinolinate, NMN and NAD^+^) and nucleotide metabolism (thymine, uracil and adenine).

To determine whether the genes correlated with a particular metabolite were enriched for specific cellular functions, we performed an unsupervised pathway enrichment analysis. For each metabolite with at least one GMI, we investigated whether specific pathways and processes were over-represented in the transcripts correlated with that metabolite. Overall, the considered transcripts spanned over 146 KEGG pathways (Supplementary Table 4), covering the 85 metabolic processes in Fig. 3 as well as cellular processes (for example, cell growth and death), signalling pathways, genetic processing pathways (for example, transcription and translation) and organismal systems pathways (for example, immune, endocrine and sensory systems). A total of 32 unique pathways were over-represented across 40 metabolites (adjusted *P* value < 0.01; Fig. 4c). Interestingly, only 2 of those 32 pathways represented metabolic processes: oxidative phosphorylation, for which the top three most associated metabolites were adenine, NMN and FAD, and the TCA cycle, also associated with FAD. The remaining top-ranked pathways were exclusively non-metabolic ones. Three metabolites in particular, quinolinate, NMN and NAD^+^, showed broad enrichment for immune-related cellular processes, including chemokine signalling, as well as B cell and T cell antigen receptor signalling pathways. This indicates that these metabolites are correlated with the expression of a wide array of genes associated with the immune response.

### NAD^+^-related metabolites associate with immune cell infiltration

Human tumour tissues are heterogeneous compositions of various cell populations, including tumour cells, immune cells and stromal cells. Bulk and single-cell profiling technologies have established that a large subset of genes are exclusively expressed in immune cells or non-immune cell subpopulations (for example, tumour cells)^44,45^. We reasoned that the correlation between NAD^+^-related metabolites and immune-related genes (Fig. 4c) could therefore arise if NAD^+^-related metabolites were at a characteristically higher or lower abundance in immune cells relative to non-immune cells. One implication of this hypothesis is that, while each cancer type might demonstrate its own unique metabolomic changes associated with immune infiltration, NAD^+^-related metabolites should be expected to demonstrate consistent effects across many different cancer types.

**Fig. 5:**
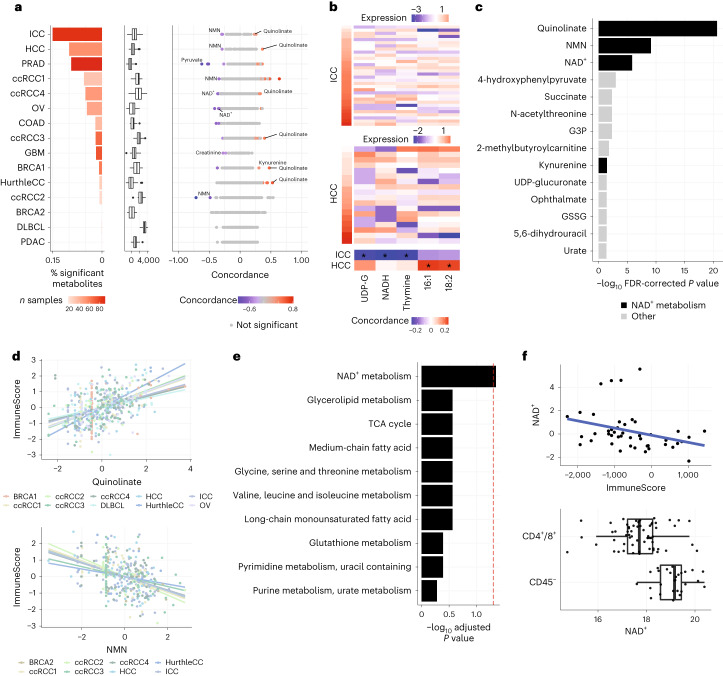
The abundance of NAD^+^-related metabolites is shaped by immune infiltration in the tumour microenvironment. **a**, Left, association of metabolite abundance with immune infiltration varied significantly across cancer types. The bar plot indicates the fraction of metabolites significantly associated with the ImmuneScore signature in each dataset. Bar colours code for the sample size of the dataset. Sample size is the number of tumour samples of the dataset in Fig. 1a. Middle, box plots indicate the expression range of the ImmuneScore signature within each dataset. Right, plot of scaled concordance calculated between metabolites and ImmuneScore. Red dots indicate metabolites with a positive association with ImmuneScore, blue dots indicate metabolites with negative associations, and grey dots indicate metabolites that were not statistically significant. **b**, Heat maps of five metabolites—three that were highly correlated with ImmuneScore in the ICC dataset and two that were highly correlated with ImmuneScore in the HCC dataset. Samples were sorted by increasing ImmuneScore. UDP-G, UDP-glucuronate; GPC-16:1, 1-palmitoleoyl-GPC (16:1); GPC-18:2, 1-linoleoyl-GPC (18:2). **c**, Bar plot indicating the strength of association between metabolites and ImmuneScore from concordance meta-analysis. Two-tailed *P* values were estimated from the unscaled concordance value’s *z*-score (Methods) and were corrected for multiple testing using the Benjamini–Hochberg method. Bar length represents the −log_10_ adjusted *P* value. Metabolites related to NAD^+^ metabolism are shown in black. **d**, Scatterplots of the abundance of two NAD^+^-related metabolites, quinolinate and NMN, versus ImmuneScore expression across all datasets. Metabolite abundances were scaled within each dataset. **e**, Bar plot comparing the absolute concordance values of metabolites to ImmuneScore in a pathway compared to all other pathways (one-sided *P* values were estimated from Wilcoxon rank-sum test and were corrected for multiple testing using the Benjamini–Hochberg method). **f**, Metabolomic measurements of purified populations of CD45^−^ tumour cells and CD45^+^ T cells isolated from ovarian cancer tumours. NAD^+^ was negatively correlated with the ImmuneScore signature in the ovarian cancer dataset (*n* = 45). NAD^+^ was similarly lower in abundance in CD4^+^/CD8^+^ cells than CD45^−^ (non-immune) cells in the dataset of purified cell populations (*n* = 24 in CD4^+^/CD8^+^; *n* = 18 in CD45^−^). Source data

To determine if NAD^+^-related metabolites were associated with immune infiltration across tumours, we used single-sample gene-set enrichment analysis (ssGSEA)^46^ to compute a previously validated 141-gene RNA signature of overall immune cell infiltration (ImmuneScore) directly from bulk RNA-seq data^47^, and identified the individual metabolites correlated with this immune phenotype. Concordance between metabolite levels and the ImmuneScore signature was assessed across all samples in each CAMP dataset (Fig. 5a). In general, covariation between specific metabolite pools and ImmuneScore expression was cancer-type specific. For example, of the metabolites significantly associated with ImmuneScore in intrahepatic cholangiocarcinoma (ICC) and hepatocellular carcinoma (HCC; representing the top two cancer types with the highest number of metabolites significantly associated with ImmuneScore), only 3 metabolites were consistently associated with ImmuneScore in both datasets (Fig. 5b and Supplementary Table 5). We did not observe a significant correlation (Spearman’s rank correlation *P* value = 0.69) between the percentage of metabolites significantly associated with ImmuneScore and expression of the ImmuneScore signature itself (Fig. 5a), suggesting that the extent of immune infiltration did not confound our analyses.

While metabolite concordance with ImmuneScore was generally heterogeneous across CAMP datasets, we observed that several NAD^+^-related metabolites, including quinolinate and NMN, were recurrently associated with high or low levels of immune infiltration in numerous disease contexts (Fig. 5a). To systematically identify such lineage-agnostic metabolomic correlates of immune infiltration, we again applied concordance meta-analysis, identifying 14 metabolites significantly associated with ImmuneScore across datasets (adjusted *P* value < 0.05; Fig. 5c), with quinolinate and NMN being the strongest hits (Fig. 5d). Interestingly, 4/14 significantly associated metabolites (quinolinate, NAD^+^, NMN and kynurenine) were members of the NAD^+^ biosynthesis pathway. Consistent with this, we identified NAD^+^ metabolism as the sole pathway whose metabolites demonstrated significantly stronger association with immune infiltration than all other pathways (adjusted *P* value = 0.04; Fig. 5e).

The above analysis suggested that NAD^+^-related metabolites were at differential abundance in immune cells relative to non-immune cells, and that this effect produced a consequent accumulation of NAD^+^-related metabolites in immune-infiltrated tumours. Some support for this hypothesis can be found in previously published immunohistochemical data indicating that the abundance of quinolinate increases dramatically in diverse immune cell populations in response to Toll-like receptor 4 ligands such as lipopolysaccharide^48^. To provide more evidence for this hypothesis, we compared our findings to a recently published study of metabolomic profiles of purified CD45^−^ tumour cells and CD45^+^ (CD8^+^ and CD4^+^) T cells from ovarian cancer tumours^48^. In our data, NAD^+^ was negatively correlated with ImmuneScore in ovarian cancer, suggesting that it was at lower abundance in immune cells relative to non-immune cells (Fig. 5f). Consistent with this, NAD^+^ was at significantly lower abundance in CD45^+^ T cells than CD45^−^ tumour cells in the dataset of purified cell populations (NAD^+^ log_2_ fold change = −1.22, *P* value = 1.7 × 10^−^^4^; Fig. 5f). Together, these analyses suggest that the pool sizes of NAD^+^-related metabolites are at characteristically different abundance in immune cells relative to other cell types, and that this effect ultimately drives the association of quinolinate and other NAD^+^-related metabolites with immune infiltration in bulk tumour data.

### Kynurenine and histamine levels correlate to specific cell populations

**Fig. 6:**
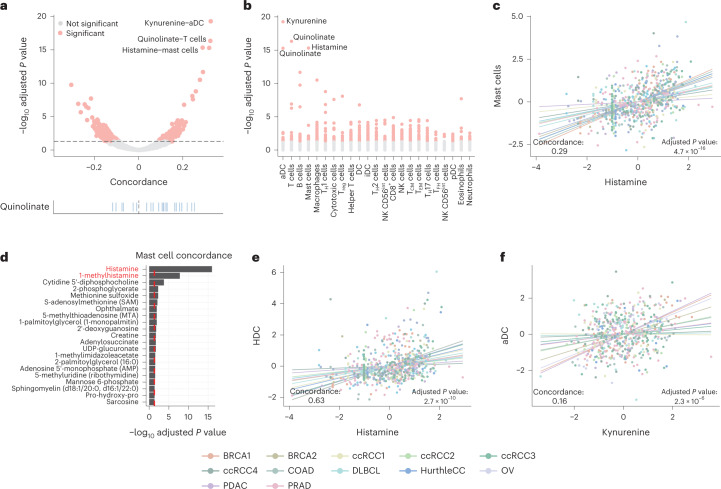
A subset of metabolites associates with specific immune cell lineages. **a**, Volcano plot of cell-type signature–metabolite interactions. The rug plot at the bottom highlights the numerous associations with quinolinate. **b**, Manhattan plot of adjusted concordance *P* values between metabolites and cell types. **c**, Histamine associates with the abundance of mast cells across most datasets of the CAMP. **d**, Bar plot of adjusted concordance *P* values of metabolites correlated with the mast cell gene signature. The red dashed line indicates the significance cut-off of 0.05. Metabolites labelled in red are associated with histamine metabolism. **e**, *HDC* expression strongly associates with mast cell abundance across the CAMP. **f**, Kynurenine abundance associates with an aDC signature across the CAMP. In **a**–**f**, two-tailed *P* values were estimated from the *z*-scores of the unscaled concordance values (Methods) and were corrected for multiple testing using the Benjamini–Hochberg method. Source data

Whereas in the previous analysis we investigated the association of metabolite levels with overall immune infiltration, we next turned to investigating the association of metabolite levels and specific immune cell populations (for example, T cells, macrophages and numerous other cell types), each with unique transcriptional phenotypes and immunological functions^49–51^. To investigate how these diverse immune cell populations contributed to the observed GMIs, we estimated the abundance of 23 immune cell types from bulk transcriptomics profiles^51^ using ssGSEA as shown previously^46^, and computed their association with metabolite levels across cancers. We again focused on lineage-agnostic relationships by performing a concordance meta-analysis to calculate associations between metabolite levels and immune cell signatures across tumours from all cancer types. In total, 7.3% of all metabolite–signature pairs (466 of 6,348 pairs) demonstrated statistically significant associations (adjusted *P* value < 0.05; Fig. 6a). Among these, quinolinate was positively associated with almost all immune cell populations (17/23), consistent with prior immunohistochemical data and suggesting that it accumulates in a variety of immune cell types in a cancer-type-agnostic manner^52^.

**Extended Data Fig. 7:**
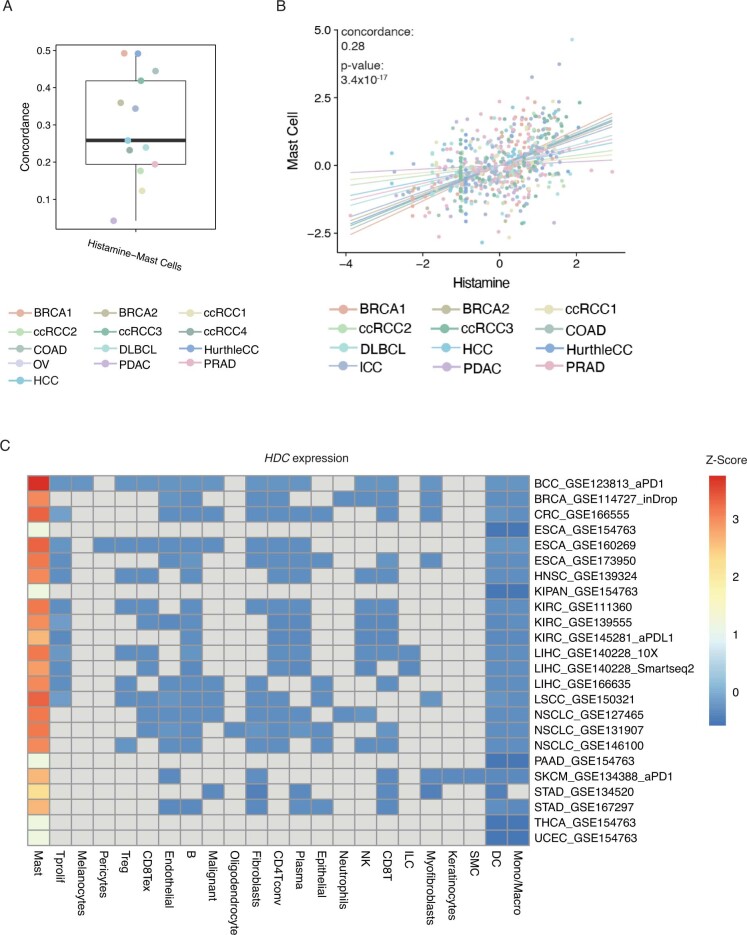
Diverse cancer types likely drove the association between histamine and mast cells. (A) Box plots of concordance of histamine – mast cells across 13 datasets. The data is displayed as a boxplot, in which the box bounds represent the 25th to the 75th percentile, with the median shown as a line in the center. The bottom whisker extends to the smallest value that is not an outlier, and the upper whisker extends to the largest value that is not an outlier. (B) Scatterplots comparing expression of the mast cell signature without *HDC* to histamine abundance. Two-tailed p-values were estimated from the unscaled concordance value’s z-score (see Methods). (C) Heatmap of single cell gene expression data for *HDC* across datasets from the TISCH2 database^85,86^. Expression is standardized to z-scores across cell types in a dataset. Considering only datasets in which mast cells are identified, mast cells exhibit highest expression of *HDC*. Source data

Aside from quinolinate, the two strongest associations between immune cell-type signatures and metabolite levels were two comparatively rare cell populations, namely mast cells and histamine and activated dendritic cells (aDCs) and kynurenine (Fig. 6b). Mast cells are a myeloid cell population that, when stimulated, mediate the inflammatory process by synthesizing histamine from histidine using the enzyme HDC. We found both histamine and its related metabolite 1-methylhistamine were the two metabolites most significantly associated with the presence of mast cells (Fig. 6c,d), and that the association between histamine and mast cell abundance was driven by diverse cancer types in the CAMP (Extended Data Fig. 7a). Histamine levels themselves were strongly associated with the expression of *HDC* across the CAMP (Fig. 6e). Importantly, the mast cell signature contains *HDC*, but when the signature was recalculated without *HDC* the significant positive concordance was preserved (concordance = 0.28, *P* value = 3.4 × 10^−^^17^; Extended Data Fig. 7b). Moreover, single-cell data also indicate that *HDC* expression is strongly elevated in mast cells relative to other cell types (Extended Data Fig. 7c). Interestingly, the median variation of histamine across the tumour datasets in the CAMP was ~740-fold, implying that fluctuations in the abundance of an otherwise rare cell population were sufficient to produce large-magnitude changes in histamine abundance in the bulk tumour.

**Extended Data Fig. 8:**
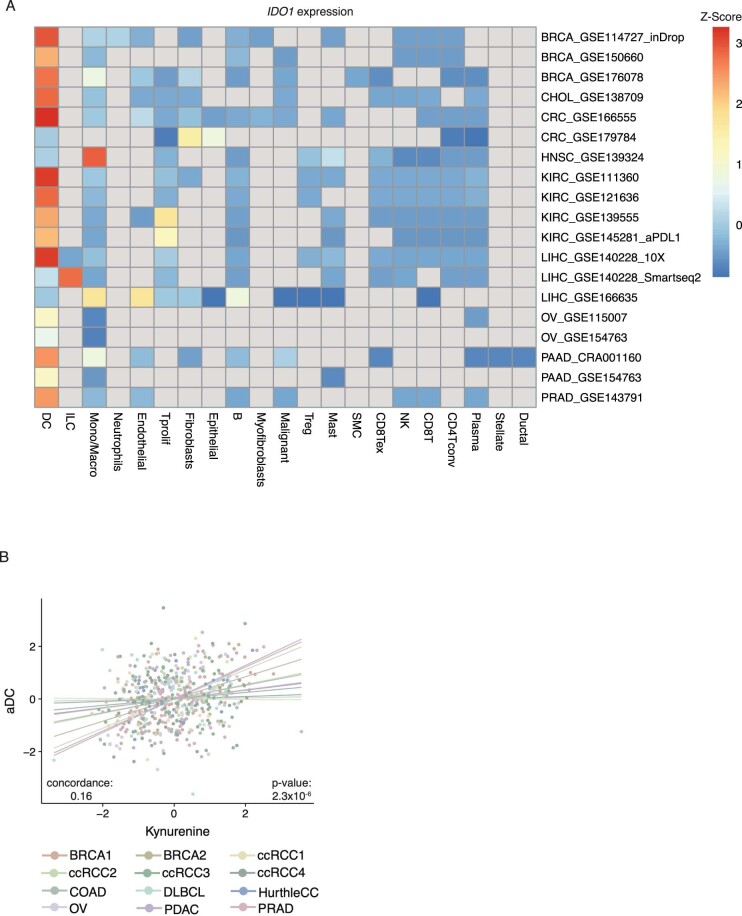
The association between kynurenine and dendritic cells was not solely dependent on *IDO1* itself. (A) Heatmap of single cell gene expression data for *IDO1* across datasets from the TISCH2 database^85,86^. Expression is standardized to z-scores across cell types in a dataset. Considering only datasets in which dendritic cells are identified, dendritic cells exhibit the highest expression of *IDO1*. (B) Scatterplots comparing expression of the aDC signature without *IDO1* to kynurenine abundance. Two-tailed p-values were estimated from the unscaled concordance value’s z-score (see Methods). Source data

In contrast to mast cells and their physiological role in producing histamine, DCs are not known to be dedicated sources of kynurenine in the microenvironment, although single-cell data indicate that *IDO1* expression is strongly elevated in DCs relative to other cell types (Extended Data Fig. 8a). While the aDC signature contains *IDO1* (and *IDO1* participates in a strong GMI with kynurenine; Fig. 2), an aDC signature without *IDO1* preserved strong positive concordance with kynurenine (concordance = 0.16, *P* value = 3.3 × 10^−^^5^; Extended Data Fig. 8b), confirming that this association was not solely dependent on *IDO1* itself (Fig. 6f). *IDO1* and kynurenine corresponded to the strongest GMI in the CAMP, raising two opposing hypotheses on the mechanism underlying the primary source of kynurenine in the tumour microenvironment: either bulk tumour expression of *IDO1* is primarily determined by high DC-specific expression or, alternatively, it is driven by a comparatively low expression in far more abundant non-DC-cell populations. Resolving this ambiguity would require single-cell measurements of metabolite levels, which have recently become technically feasible^53^. Together, these data suggest that the presence of individual and comparatively rare cell populations is associated with shifts in the abundance of immunomodulatory metabolites in the tumour microenvironment.

## Discussion

Metabolism is jointly controlled by genetically encoded enzymes and small-molecule metabolites. To study the interactions between genes and metabolites at scale, we assembled and harmonized a database of metabolomic and transcriptomic data from ~1,000 tumour and normal samples, which we refer to as the CAMP. Although large-scale multimodal measurements of metabolism have previously been produced in bacteria^54^ and yeast^55^, a comparable resource for human cancers was missing. The CAMP is thus a resource and represents a significant public database for studying the metabolism of complex human tissues and cancers, and for the interrogation of gene–metabolite covariation across different tissue and disease contexts. Our analysis of the CAMP demonstrates that large-scale studies of multimodal metabolic data can reveal fundamental principles of metabolic regulation at the scale of both individual metabolic reactions (Fig. 2) and complex human tissues (Figs. 5 and 6).

Our detailed statistical analysis of the CAMP revealed a multitude of gene–metabolite associations that transcended the tissue of origin. We focused on two specific types of gene–metabolite covariation. The first, induced by functional proximity, identified metabolic genes whose activation or inhibition is likely to have a significant effect on the direct substrate or product of the respective reaction (Fig. 2). While metabolite pools are likely controlled by a large number of genes and other factors, our data-driven analysis identified a small subset of metabolites whose pool size was strongly associated with a single, proximal gene across tissue lineages. The GMIs identified in Fig. 2b offer a data-informed, rational approach for modulating the pool sizes of their metabolite constituents. While each of the highlighted metabolites in Fig. 2b may participate in numerous metabolic reactions, data from the CAMP specifically nominates single genes (for example, *GGT1* for GSSG, and *IDO1* for tryptophan and kynurenine) as those targets whose perturbation is most likely to disrupt the corresponding pool size.

The second broad form of gene–metabolite covariation, likely induced by cell-type-specific physiology, corresponded to metabolites whose abundance was associated with the presence of specific immune cells in the microenvironment. Thus, a sizeable fraction of non-proximal GMIs was associated with a small number of metabolites (enriched for NAD^+^-related molecules) that were significantly correlated with a large number of genes, and in particular immune-related genes. This apparent association between the abundance of specific immune cells in a tissue specimen and the levels of numerous NAD^+^-associated metabolites suggests that immune cells have evolved mechanisms to maintain the concentrations of these metabolites at characteristically different levels relative to other cell types. Because NAD^+^ is both the central mediator of redox poise in the cell and a cofactor for numerous metabolic and non-metabolic reactions, understanding the mechanisms by which immune cells achieve differential abundance of NAD^+^-related metabolites, and the selective pressure to do so, can provide insights into the metabolic phenotypes underlying both cancer and other diseases involving dysfunctional immune responses^56^. Emerging spatial metabolomics (for example, high-resolution MALDI) and single-cell metabolomics (for example, rapid purification with paramagnetic beads, followed by mass spectrometry) technologies hold the promise of revealing the full extent of cell-type-specific metabolomic adaptations. Such approaches can be used alone or in tandem to measure the metabolomic profiles of individual cells in heterogeneous tissue slices^57–60^, rendering the full extent of cell-type-specific metabolomic alterations within experimental reach.

Our findings here nominate a new paradigm for understanding tumour-associated changes in the metabolome and how it relates to the cellular composition of the tissue microenvironment. Our discovery that the abundances of NAD^+^-related metabolites change consistently across tissues as a function of immune cell abundance adds to a limited but pre-existing understanding of how cell-type-specific metabolism enables function (for example, in the accumulation of lipids in adipocytes and clear-cell tumour cells). More importantly, the discovery that the tumour metabolome changes significantly based on immune cell composition has significant implications for the interpretation of metabolomic data in the context of cancer. Prior studies (including those from our own group) have often used adjacent normal tissue as a reference to understand tumour-specific changes in metabolite levels. What the discoveries here reinforce is that changes in the bulk tumour metabolome may not be caused by tumour-cell-intrinsic changes in metabolism, and instead may in some or many cases arise from non-malignant cell populations.

Discoveries about the function of cancer genes have emerged from a combination of untargeted, population-scale genomic surveys^61^ and mechanistic experiments in specific disease and genetic backgrounds^62^. Combining these approaches has proven transformative for the discovery of recurrent and large-effect-size alterations and prompted their characterization in model systems of human disease. In contrast, the field of cancer metabolism has primarily been driven by bottom-up experiments with modest support from large-scale (largely genetic or transcriptomic) datasets^63–65^. The CAMP is a counterpoint to these efforts. By assembling and harmonizing in one database metabolomic and transcriptomic data from diverse diseases, the CAMP represents a unique opportunity for de novo discovery of translationally relevant metabolic phenotypes in cancer. Expanding the scope of the CAMP to include both additional multimodal metabolic data as it is published (enabled by open-source code; Data availability and Code availability) and other forms of data, including but not limited to genomic sequencing, epigenetic profiling and proteomic measurements, holds the potential to reveal entirely new and highly recurrent metabolic phenomena in cancer.

## Methods

### Collection of Cancer Atlas of Metabolic Profiles

We combined 12 published datasets with 3 additional in-house datasets that profiled metabolite and gene expression from the same samples to create a comprehensive collection of 988 samples (764 tumour samples and 224 adjacent normal samples) across 11 different cancer types, covering 15 datasets, which we called the CAMP. Details and references associated with these studies are provided in Table 1 and Fig. 1a. Data are available for download at 10.5281/zenodo.7150252.

### Gene expression data processing pipeline

Six of the CAMP datasets included gene expression data captured by the Affymetrix platform (GSE28735, GSE37751, GSE26193, GSE62452, GSE76297 and Cornell_PROSTATE). For these datasets, CEL files were downloaded from the Gene Expression Omnibus or from their source within our respective institutions. Then, we applied the robust multichip average (RMA) algorithm for background subtraction, quantile normalization and summarization (via median-polish) by using the ‘rma’ function implemented in the R oligo package (version 1.40.2) (ref. ^66^). Each dataset’s rma-normalized expression matrix was then used for downstream analysis. In the COAD dataset, gene expression data were captured by an Agilent custom array, and we downloaded the gene expression matrix deposited at the Gene Expression Omnibus repository (GSE89076) for further downstream analysis.

For CAMP datasets with RNA-seq data, RNA-seq reads were aligned against human genome assembly hg19 by STAR 2-pass alignment^67^ (version 2.5.3a). QC metrics, such as general sequencing statistics, gene feature and body coverage, were then calculated based on the alignment result through RSeQC^68^ (version 2.6.4). RNA-seq gene-level count values were computed by using the R package GenomicAlignments^69^ (version 1.14.2) over aligned reads with UCSC KnownGene^70,71^ with hg19 as the base gene model. The Union counting mode was used, and only mapped paired reads after alignment quality filtering were considered. Finally, gene-level TPM (transcripts per million) and raw read count values were computed by the R package DESeq2 (version 1.18.1) (ref. ^72^). All concordance analysis described herein with RNA-seq data used TPM values for RNA-seq data.

**Extended Data Fig. 9:**
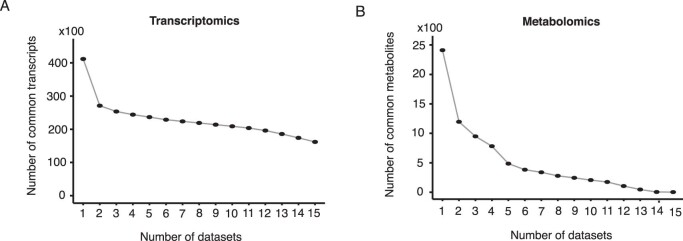
Number of common transcripts and metabolites in tumor tissue in the CAMP dataset. (A) Number of common transcripts across datasets in tumor tissue. (B) Number of common metabolites across datasets in tumor tissue. Source data

Across the 15 CAMP datasets, 16,082 of ~40,000 distinct transcripts were profiled in all cohorts and used for analysis (Extended Data Fig. 9a).

### Metabolomics data preprocessing

**Extended Data Fig. 10:**
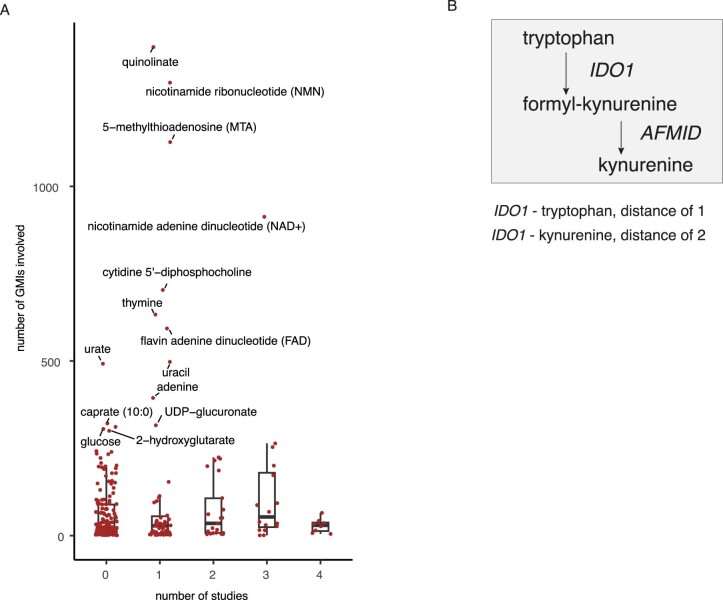
Nearly all metabolites with a large number of GMIs were imputed in 2 or fewer studies, and the definition of proximal distance. (A) Boxplot of number of studies in which the metabolite is imputed in 50% or more of the samples from 269 metabolites involved in the 22619 GMIs. The data is displayed as a boxplot, in which the box bounds represent the 25th to the 75th percentile, with the median shown as a line in the center. The bottom whisker extends to the smallest value that is not an outlier, and the upper whisker extends to the largest value that is not an outlier. (B) Definition of “proximal” interactions in the Human 1 metabolic network model, illustrated by the example of *IDO1* distances to tryptophan and kynurenine. Source data

Metabolomics data for 3/15 datasets (GBM, LiCa1 and LiCa2) were provided preprocessed and were therefore used in their original form. Ovarian cancer data were already normalized and were only log_2_ transformed before analysis. For the other 11 metabolomics datasets in our study, we obtained the raw metabolomics data from the data owners. In this case, the processing pipeline was standardized across datasets and included batch correction via median scaling if multiple batches of data were present (only necessary for PRAD dataset as it was the only dataset produced in distinct batches) and probabilistic quotient normalization^73^ using either only normal samples if available, or all tumour samples if no normal sample was included, and only metabolites with less than 20% values missing to create the reference sample. Importantly, nearly all metabolites with a large number of GMIs were imputed in two or fewer studies (Extended Data Fig. 10a). After normalization, metabolite abundances were log_2_ transformed. For each cohort and tissue type (that is, tumour and normal), metabolites with more than 80% values missing were excluded from the analysis. For the remaining metabolites, missing values were imputed using the minimum value recorded. Data preprocessing was performed using the R package maplet. Metabolite names and annotations were manually harmonized for consistency. First, we identified potentially matching compounds by systematically investigating the HMDB^74^, KEGG^75^ and Metabolon platform-specific peak IDs across datasets. The resulting candidates for each query were then manually investigated to make sure they corresponded to the same molecule. Incorrectly aggregated metabolites were discarded, and a single metabolite name was chosen to represent all validated matching compounds. The overlap of metabolites across datasets was heterogeneous (Extended Data Fig. 9b): of 2,411 unique molecules quantified in at least 1 dataset, fewer than 500 were measured in more than 5 datasets, and only 3 metabolites (gluconate, glucose and glucose-3-phosphate) were quantified in all 15 datasets in tumour samples. This high variability can be attributed to a variety of technical and biological factors, including metabolite ionizability on the mass spectrometer and potential specificity of certain metabolites to distinct tissues. A detailed, step-by-step tutorial describing how to harmonize additional future metabolomics datasets can be found in the Zenodo repository (10.5281/zenodo.7150252, supplementary_dataset_v0.3.4.zip).

### Concordance meta-analysis

To identify gene–metabolite pairs that were consistently associated across tumour types and cohorts, we used a stratified, weighted concordance model. Concordance is a non-parametric measure of correlation, similar to Kendall’s tau, that relies on the concept of concordant pairs^37^.

Briefly, consider sample $i$ and sample $j$ in a dataset where both $m$ (metabolite) and $g$ (gene) have been measured. The pair of sample *i* and sample *j* is defined to be concordant if ${sign}({m}_{i}-{m}_{j})={sign}({g}_{i}-{g}_{j})$, that is, if they have the same order in both samples, and discordant if they have opposite signs. Once pairwise concordance has been estimated for all pairs, the overall concordance *c* is calculated as

$$c=\frac{{\rm{\#}}{concordant\; pairs}+{\rm{\#}}{tied\; pairs}}{{\rm{\#}}{concordant\; pairs}+{\rm{\#}}{discordant\; pair}s+{\rm{\#}}{tied\; pairs}}$$

To account for the multiple cohorts in our study, we performed a stratified concordance analysis, where pairwise comparisons are only calculated within datasets, but not across. A global concordance value is then estimated by counting the overall number of concordant pairs according to the formula above. Moreover, given the vast heterogeneity in sample sizes across studies, we downweighed each observation by the number of samples in the corresponding study (that is, $1/{samples}({{dataset}}_{i})$), so that each dataset would contribute equally to the overall concordance. The concordance calculation was performed using the concordance function from the survival R package (v3.2-3) (ref. ^76^).

To make this quantity more intuitive, we further scaled the concordance range to values between −1 and 1 through *c*_scaled_ = 2*c* − 1*,* which is analogous to Somers’ D^77^. Furthermore, in the absence of ties, this value is also identical to Kendall’s rank correlation coefficient tau^77^. In the figures presented in this paper, a scaled concordance of 0 indicates absence of association, a value greater than 0 indicates positive association, while a value less than 0 indicates negative association. As with Somers’ D and Kendall’s tau, the magnitude of *c*_scaled_ captures the strength of the effect, with values near –1 and 1 corresponding to strong discordance and concordance, respectively.

A *z*-score was computed as unscaled concordance (that is, in the range of 0–1) minus 0.5 and divided by the square root of the variance, and the resulting value was used to derive a two-tailed *P* value^78,79^. *P* values were corrected for multiple testing using the Benjamini–Hochberg method to control the FDR^80^. Code to reproduce this analysis is available in the associated GitHub repository in the 5_Concordance.R script.

For all GMI analysis (for example, relating to Fig. 2) and concordance analysis relating metabolite levels to gene expression signatures (for example, in Figs. 5 and 6), only tumour samples were used.

### Generation of *IDO1* knockout HCT116 cell line

sgRNAs (oligonucleotide sequences are indicated in Supplementary Table 6) targeting *IDO1* as well as non-targeting control were cloned into lentiCRISPRv2 puro plasmid (Addgene, 98290). Lentiviral packaging vectors psPAX2 (Addgene, 12260), pMD2.G (Addgene, 12259), along with sgRNA expressing vector were transfected into HEK293T cells using polyethylenimine transfection reagent. Around 72 h after transfection, supernatant containing lentivirus was harvested and filtered through a Whatman filter (Fisher Scientific) with a pore size of 0.45 µm to remove cell debris. Target cells (HCT116, human colon cancer cell line, American Type Culture Collection, CCL-247) were transduced with lentivirus using 8 µg ml^−1^ polybrene (Sigma). Around 72 h after lentivirus transduction, 3 µg ml^−1^ puromycin (Thermo Fisher) was added to cell culture medium to select for virus-infected cells. Two weeks after puromycin selection, target gene knockout was confirmed by western blot. For induction of *IDO1* gene expression, cells were treated with 100 ng ml^−1^ interferon-γ for 48 h.

### Metabolomics profiling on the *IDO1* knockout HCT116 cell line

Cells were plated in six-well tissue culture plates at a density of 100,000 cells per well. Around 72 h after cell seeding, metabolites were extracted and analysed by liquid chromatography–mass spectrometry (LC–MS). For metabolite extraction, culture medium was aspirated, and cells were washed once with ice-cold PBS. After PBS washing, 1 ml of ice-cold extraction solvent (methanol:water ration of 80:20) was added. After overnight incubation at −80 °C, cells and extraction solvent were collected into 1.5-ml microcentrifuge tubes using a cell scraper. Samples were centrifuged at 20,000*g* for 20 min at 4 °C. Supernatant (900 μl) was collected and dried in a vacuum evaporator (Genevac EZ-2 Elite).

For LC–MS, dried extracts were resuspended in 30 μl of a 97:3 ratio of water:methanol containing 10 mM tributylamine and 15 mM acetic acid. Samples were vortexed, incubated on ice for 20 min, and clarified by centrifugation at 20,000*g* for 20 min at 4 °C. LC–MS analysis used a Zorbax RRHD Extend-C18 column (150 mm × 2.1 mm, 1.8-μm particle size, Agilent Technologies). Solvent A was 10 mM tributylamine, 15 mM acetic acid in a 97:3 ratio of water:methanol, and solvent B was 10 mM tributylamine and 15 mM acetic acid in a 3:97 ratio of water:methanol, prepared according to the manufacturer’s instructions (MassHunter Metabolomics dMRM Database and Method, Agilent Technologies). LC separation was coupled to a 6470 triple-quadrupole mass spectrometer (Agilent Technologies), which was operated in dynamic MRM scan type and negative ionization mode.

### Tumour versus normal pathway analysis

There were seven CAMP datasets that had both tumour and normal samples available (BRCA1, COAD, GBM, PRAD, PDAC, ccRCC3 and ccRCC4). We applied differential gene expression tests between tumour and normal samples in each dataset using limma-voom (limma package, version 3.5.2). Genes with an FDR-adjusted *P* value < 0.1 were considered significantly differentially expressed. Similarly, we also conducted differential metabolite abundance testing between tumour and normal samples using Wilcoxon rank-sum tests. Metabolites with an FDR-adjusted *P* value < 0.1 were considered significantly differentially abundant.

For each KEGG pathway, we calculated the DF score and the DA score for genes and metabolites separately:

DF score = (number of significantly up-regulated constituents + number of significantly down-regulated constituents) / number of measured constituents in a pathway

DA score = (number of significantly up-regulated constituents − number of significantly down-regulated constituents) / number of measured constituents in a pathway

Constituents are either genes or metabolites in the above formula.

Conceptually, the DF score captures the overall disruption of the constituents of a pathway, whereas the DA score captures the tendency for pathway constituents to increase or decrease in abundance relative to a reference (in this case, normal tissue) state.

### Gene–metabolite distance

To define a distance between genes and metabolites, we considered the highly manually curated genome-scale human metabolic model from Robinson et al.^41^, referred to as the ‘Human-1’ model, which describes the metabolic reaction network of transporters, enzymes and metabolites. We systematically computed the distance between a gene and a metabolite according to how many reaction steps separate the two molecules. If a gene and a metabolite participate in the same reaction, they will be assigned a distance of one; if they take part in subsequent reactions, they will be assigned a a distance of two, and so on. We defined an interaction as ‘proximal’ if the corresponding gene–metabolite pair had a distance of one or two (Extended Data Fig. 10b).

Overall, we could compute a distance for 473,206/4,438,632 (~10.66%) gene–metabolite pairs in our analysis, and 78,672/473,206 (~16.62%) of these were classified as proximal (that is, a distance of one or two). Of all significant gene–metabolite pairs in our concordance meta-analysis, 3,304/22,619 pairs had a defined distance (~14.61%), but only 565/22,619 (~2.50%) of these were proximal.

### Gene-set enrichment analysis

For the analysis in Fig. 4, we ran a pathway enrichment analysis among all significant genes for each metabolite with at least one significant gene association. This set of genes was mapped to a total of 146 KEGG pathways, and the enrichment test was performed using classical hypergeometric testing^81^. *P* values were adjusted using the Benjamini–Hochberg method for controlling the FDR^80^. Adjusted *P* values < 0.01 were considered significant.

Results were then aggregated into a metabolite × pathway matrix and visualized as a heat map, where metabolites and pathways were clustered based on the –log_10_(*P* value). Clustering was performed using the pheatmap function^82^ (pheatmap package, v1.0.12) with Ward linkage and Euclidean distance.

### Bulk gene expression deconvolution analysis

To dissect the role of the immune compartment in the tumour microenvironment, we calculated the ImmuneScore through the estimate R package^83^. To calculate cell-type-specific infiltration patterns in Fig. 6, we used ssGSEA^46^ for bulk gene expression deconvolution analysis. Signature gene lists of immune cell types and immune features were obtained from Bindea et al.^51^. Briefly, ssGSEA takes the sample TPM expression values as the input and computes an enrichment score for a given gene list as compared to all the other genes in the sample transcriptome.

### Statistics and reproducibility

No statistical method was used to predetermine sample size. No data points were excluded from the analyses. The statistical tests used for the individual analyses are indicated in the figure legends. The experiments were not randomized. The investigators were not blinded to allocation during experiments and outcome assessment.

### Reporting summary

Further information on research design is available in the Nature Portfolio Reporting Summary linked to this article.

## Supplementary information

Reporting Summary

Supplementary Tables 1–6 **Supplementary Table 1** Table of statistically significant GMIs and associated statistics. Two-tailed *P* values were estimated from the unscaled concordance value’s *z*-score (Methods) and were corrected for multiple testing using the Benjamini–Hochberg method. **Supplementary Table 2** Summary of DA results for pathways considered in tumour versus normal analysis. **Supplementary Table 3** Association of DF gene/metabolite scores across KEGG pathways in tumour versus normal analysis. *P* values were estimated from Spearman’s rank correlation test and were corrected for multiple testing using the Benjamini–Hochberg method. **Supplementary Table 4** Pathways considered for the analysis in Fig. 4. **Supplementary Table 5** Comparison of metabolite correlated to ImmuneScore in the HCC and ICC datasets. Two-tailed *P* values were estimated from the unscaled concordance value’s *z*-score (Methods) and were corrected for multiple testing using the Benjamini–Hochberg method. **Supplementary Table 6** Guide RNA sequences used for *IDO1* knockout experiment.

## Data Availability

All data needed to evaluate the conclusions in the paper are present in the paper and/or the Supplementary Information. Processed metabolomics and RNA-seq data are publicly available at 10.5281/zenodo.7150252. A data portal is available as an online Shiny app (https://rezniklab.shinyapps.io/CAMP-shiny-app/). Source data are provided with this paper.

## Source data

Source Data Figs. 1–6 Statistical source data.
Source Data Fig. 2f Unprocessed blots and gels.
Source Data Extended Data Figs. 1–10 Statistical source data.

## Code availability

R code with instructions to generate relevant analysis and figures is available from GitHub (https://github.com/reznik-lab/pancancer-metabolomics/; https://github.com/reznik-lab/CAMP-shiny-app/) and under Zenodo (10.5281/zenodo.7150252). Users can introduce new datasets by following the steps in the Zenodo repository (10.5281/zenodo.7150252, supplementary_dataset_v0.3.4.zip).
