# Supplementary material for: A multimodal atlas of tumour metabolism reveals the architecture of gene–metabolite covariation
Source: Nat Metab. 2023 Jun 19;5(6):1029–44. doi: 10.1038/s42255-023-00817-8 (PMC10290959; doi:10.1038/s42255-023-00817-8)
Supplement: Supplementary file 1 — Reporting Summary [file 42255_2023_817_MOESM1_ESM.pdf]

nature portfolio

Corresponding author(s): Jan Krumsiek, Ed Reznik

Last updated by author(s): Apr 14, 2023

Reporting Summary

Nature Portfolio wishes to improve the reproducibility of the work that we publish. This form provides structure for consistency and transparency in reporting. For further information on Nature Portfolio policies, see our Editorial Policies and the Editorial Policy Checklist.

Statistics

For all statistical analyses, confirm that the following items are present in the figure legend, table legend, main text, or Methods section.

n/a	Confirmed	
☐	☒	The exact sample size (*n*) for each experimental group/condition, given as a discrete number and unit of measurement
☐	☒	A statement on whether measurements were taken from distinct samples or whether the same sample was measured repeatedly
☐	☒	The statistical test(s) used AND whether they are one- or two-sided<br>*Only common tests should be described solely by name; describe more complex techniques in the Methods section.*
☒	☐	A description of all covariates tested
☐	☒	A description of any assumptions or corrections, such as tests of normality and adjustment for multiple comparisons
☐	☒	A full description of the statistical parameters including central tendency (e.g. means) or other basic estimates (e.g. regression coefficient) AND variation (e.g. standard deviation) or associated estimates of uncertainty (e.g. confidence intervals)
☐	☒	For null hypothesis testing, the test statistic (e.g. *F*, *t*, *r*) with confidence intervals, effect sizes, degrees of freedom and *P* value noted<br>*Give P values as exact values whenever suitable.*
☒	☐	For Bayesian analysis, information on the choice of priors and Markov chain Monte Carlo settings
☒	☐	For hierarchical and complex designs, identification of the appropriate level for tests and full reporting of outcomes
☒	☐	Estimates of effect sizes (e.g. Cohen's *d*, Pearson's *r*), indicating how they were calculated

Our web collection on statistics for biologists contains articles on many of the points above.

Software and code

Policy information about availability of computer code

Data collection	No software was used for data collection.
Data analysis	All analysis presented in the paper can be reproduced using the R code deposited in the github repository available at https://github.com/reznik-lab/pancancer-metabolomics. The code relies on several R libraries and algorithms: oligo (version 1.40.2), STAR 2-pass alignment (version 2.5.3a), RSeQC (version 2.6.4), GenomicAlignments (version 1.14.2), DESeq2 (version 1.18.1), maplet (version 1.1.2).

For manuscripts utilizing custom algorithms or software that are central to the research but not yet described in published literature, software must be made available to editors and reviewers. We strongly encourage code deposition in a community repository (e.g. GitHub). See the Nature Portfolio guidelines for submitting code & software for further information.

Data

Policy information about availability of data

All manuscripts must include a data availability statement. This statement should provide the following information, where applicable:

- Accession codes, unique identifiers, or web links for publicly available datasets
- A description of any restrictions on data availability
- For clinical datasets or third party data, please ensure that the statement adheres to our policy

All data analyzed in the paper are publicly available and downloadable from Zenodo (DOI: 10.5281/zenodo.7150252). A data portal to explore the datasets is also available at https://rezniklab.shinyapps.io/CAMP-shiny-app/.

Research involving human participants, their data, or biological material

Policy information about studies with human participants or human data. See also policy information about sex, gender (identity/presentation), and sexual orientation and race, ethnicity and racism.

Reporting on sex and gender	The manuscript conforms to SAGER guidelines. More specifically, the results do not conform to any single sex/gender, sex/gender was not considered in the study design as this was a meta-analysis of pre-existing data, and no information on sex/gender was available and so disaggregation was not possible.
Reporting on race, ethnicity, or other socially relevant groupings	N/A
Population characteristics	N/A
Recruitment	N/A
Ethics oversight	N/A

Note that full information on the approval of the study protocol must also be provided in the manuscript.

Field-specific reporting

Please select the one below that is the best fit for your research. If you are not sure, read the appropriate sections before making your selection.

☒ Life sciences ☐ Behavioural & social sciences ☐ Ecological, evolutionary & environmental sciences

For a reference copy of the document with all sections, see nature.com/documents/nr-reporting-summary-flat.pdf

Life sciences study design

All studies must disclose on these points even when the disclosure is negative.

Sample size	The study analyzes already published datasets, and therefore a sample size calculation was not applicable. By using a meta-analysis approach to the data analysis, we could leverage even smaller studies to identify statistically significant associations. Moreorver, all statistical findings were corrected for multiple testing to reduce the proportion of false positives.
Data exclusions	No data points were excluded from the analysis.
Replication	The study is based on computational and statistical analysis. Whenever possible, we tried to validate the finding either with targeted experiments, or with prior literature.
Randomization	The study analyzes already published datasets, therefore randomization of subject was not applicable.
Blinding	The study analyzes already published datasets, therefore blinding of was not possible, as the data were already labelled as cases/controls.

Reporting for specific materials, systems and methods

We require information from authors about some types of materials, experimental systems and methods used in many studies. Here, indicate whether each material, system or method listed is relevant to your study. If you are not sure if a list item applies to your research, read the appropriate section before selecting a response.

Materials & experimental systems

n/a	Involved in the study	
☒	☐	Antibodies
☐	☒	Eukaryotic cell lines
☒	☐	Palaeontology and archaeology
☒	☐	Animals and other organisms
☒	☐	Clinical data
☒	☐	Dual use research of concern
☒	☐	Plants

Methods

n/a	Involved in the study	
☒	☐	ChIP-seq
☒	☐	Flow cytometry
☒	☐	MRI-based neuroimaging

Eukaryotic cell lines

Policy information about cell lines and Sex and Gender in Research

Cell line source(s)	We purchased HCT116, human colon caner cell line from ATCC, CCL-247.
Authentication	HCT116 was not authenticated.
Mycoplasma contamination	HCT116 was tested negative for mycoplasma contamination.
Commonly misidentified lines (See ICLAC register)	No misidentified cell lines were used in this study.
