# Supplementary figures and images for: A multimodal atlas of tumour metabolism reveals the architecture of gene–metabolite covariation
Source: Nat Metab. 2023 Jun 19;5(6):1029–44. doi: 10.1038/s42255-023-00817-8 (PMC10290959; doi:10.1038/s42255-023-00817-8)

IDO1

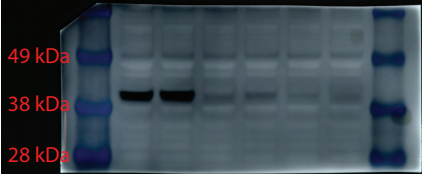

Vinculin

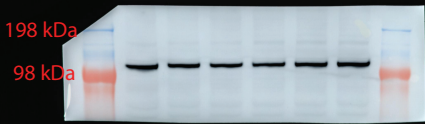

Supplement: Source Data Fig. 2f — Unprocessed blots and gels. [file 42255_2023_817_MOESM4_ESM.pdf]
